# Understanding the business versus care paradox in gambling venues: a qualitative study of the perspectives from gamblers, venue staff and counsellors

**DOI:** 10.1186/s12954-018-0256-4

**Published:** 2018-09-24

**Authors:** Ben J. Riley, Simone Orlowski, David Smith, Michael Baigent, Malcolm Battersby, Sharon Lawn

**Affiliations:** 1 Department of Health Sciences, Flinders University, Adelaide, South Australia Australia; 2 Flinders Medical Centre, Block E2, The Flats, Flinders Drive, Bedford Park, South Australia 5042 Australia; 3 Flinders University Human Behaviour Health Research Unit, Margaret Tobin Building (4T:306), GPO Box 2100, Adelaide, South Australia 5001 Australia; 4 Department of Psychiatry, Flinders University South Australia, GPO Box 2100, Adelaide, South Australia 5001 Australia; 5 Flinders Centre for Gambling Research, Flinders University South Australia, GPO Box 2100, Adelaide, South Australia 5001 Australia

**Keywords:** Problem gambling, Qualitative, Venue staff, Role conflict, Identification

## Abstract

**Background:**

In recent years, greater emphasis has been placed on gambling venues to identify potential problem gamblers, respond appropriately and refer to treatment. In seeking the perspectives of problem gamblers, venue staff and treatment providers, this qualitative study investigates how problem gamblers experience being identified and referred for treatment by venue staff.

**Methods:**

A semi-structured interview guide focusing on experiences and perceptions of problem gambling identification and referral for treatment in gaming venues was used to conduct 4 focus groups and 9 semi-structured in-depth interviews. Participants comprised 22 problem gamblers, 10 gambling venue staff and 8 problem gambling counsellors. Audio recordings were transcribed verbatim, and an interpretive phenomenological analysis was conducted.

**Results:**

‘Role conflict’ was identified as a considerable source of stress for venue staff who described conflicting priorities in responding to problem gamblers whilst maintaining employer profit margins. Problem gamblers described offers of help from venue staff as hypocritical and disingenuous. Venue staff also described reluctance to make moral judgements through the identification of and engagement with problem gamblers, and gamblers described resentment in being singled out and targeted as a problem gambler. Being approached and offered referral to a counselling service was a rare occurrence among problem gamblers. This corresponded with reports by gambling counsellors.

**Conclusions:**

Role conflict experienced by gambling venue staff and patrons alike inhibits effective referral of potential problem gamblers into treatment. Reducing the need for gambling venue staff to make a perceived moral judgement about the gambling behaviours of specific patrons may improve the reception of responsible gambling information and promote help-seeking.

## Background

Australia has the highest gambling participation rate [1] and among the largest number of electronic gaming machines per capita in the world [2]. An estimated 2.5% of Australian adults experience moderate to severe problems caused by problem gambling (PG) [1]. For every problem gambler, around six others such as partners and children are also adversely affected [3] suggesting up to four million Australians can experience emotional, social and financial stress caused by PG. Despite this, studies from Australia and the USA report only a small number of problem gamblers seek help [1, 4], and for those that do, help-seeking is often a last resort after experiencing significant negative consequences [1, 5].

Given the general reluctance of individuals with gambling problems to seek help [5–8] and reported low levels of awareness of help services among problem gamblers [8], gambling venues provide a valuable opportunity for staff to inform gamblers of available help services and offer referral. Consequently, gaming room resources and staff interactions with gamblers may have important public health implications, particularly, as venue staff are among the first point of contact for individuals looking for help with gambling problems [1]. Furthermore, there is some evidence that as gamblers develop rapport with the staff and view them as trustworthy [1]. Frontline gambling venue staff therefore could provide an important gateway to encourage problem gamblers to seek treatment and to facilitate referrals [9]. As such, there has been increased interest in the degree to which gambling venue staff can identify problem gamblers and take an active role in intervening before further harm is endured [10].

A number of studies have found venue staff feel confident in their ability to identify problem gamblers [11–13]. However, the only study to date which has examined staff accuracy in PG identification found that venue staff were able to identify only 36% of patrons experiencing problems with gambling. Many gamblers who self-reported at least moderate gambling problems were not classified as having any problems by staff. On the other hand, a number of gamblers in the ‘no risk’ category were classified by staff as having problems [14].

There have been several attempts to develop behavioural checklists to assist venue staff in identifying problem gamblers [15–17]. These studies suggest that a range of visible and non-visible cues, when occurring in combination during a visit, have a high confidence value in identifying someone as a problem gambler. However, the authors point out that the relatively low frequency that behavioural indicators are likely to occur, at the precise time a single onlooker might observe them, poses significant challenges for effectively using such indicators in practice [16]. Delfabbro et al. [10] argued that whilst staff may be able to observe potential indicators, this would require a period of continuous observation that would likely be impractical for staff to perform, given their other competing duties. These outcomes are consistent with the conclusions provided by Schellinck and Schrans [16] and Allcock [18], in that although it is theoretically possible to identify problem gamblers using a range of behavioural indicators, there are many challenges facing venue staff if they are to rely on such indicators in practice.

In addition, staff have identified role conflict and role ambiguity as significant sources of stress [19] given on the one hand they have the role of attracting patrons, whilst at the same time there is an expectation that they approach patrons of concern, which may ultimately lead to driving the patron away to another hotel [9]. A contradiction exists in that venues, as businesses, are motivated to generate profits, whilst concurrently required to discourage problematic gambling, despite the fact that in Australia, problem gamblers contribute 40% of all money put into electronic gaming machines [1]. To date, this ‘business versus care’ paradox has received little empirical attention.

With a greater emphasis being placed on gambling venues to identify and respond to potential problem gamblers, further research is needed that focuses on how venues can best respond to potential problem gamblers to effectively facilitate harm reduction. This paper aims to address this gap by presenting a qualitative analysis from a sample of problem gamblers, gambling venue staff and gambling counsellors’ perspectives on the identification and response to PG in venues.

The present research seeks to examine the help-seeking experience in the context of gambling venues, from the perspectives of problem gamblers, gambling venue staff and gambling counsellors, and how such experiences affect responding to PG in venues. Our aim is not just to describe the experience of identification and response from the perspective of gamblers and staff, but to understand the phenomena at a deeper level [20], taking into account the various challenges, such as stigma and role ambiguity, reported by previous research. This study aims to understand this phenomenon and provide suggestions to improve effective engagement of problem gamblers in the venue. Based on these aims and objectives, the following research questions were developed for this study:

- What is the lived experience and meaning to be a gambling venue staff member in a climate of responsible gambling?
- What is the lived experience and meaning to be a problem gambler in a gambling venue in a climate of responsible gambling?
- What are the perspectives of PG counsellors concerning policies that encourage them to engage with gambling venues?
- How can gambling venues effectively facilitate help-seeking among problem gamblers?

Qualitative research is particularly helpful in providing rich descriptions of complex phenomena [21]. The primary method used in this study was interpretive phenomenological analysis (IPA) as described by Smith and Osbourne [22] utilising a Heideggerian philosophical perspective. IPA is concerned with examining how individuals make sense of their experiences [23]. Heideggerian phenomenology views that our experience always occurs and is made sense of within a situated context [24]. Therefore, an experience cannot be simply lifted from an individual’s consciousness. Rather, to understand the meaning of an experience to an individual, the researcher must engage and interpret the individual as they themselves interpret and make sense of their own experience. This two-staged analytical process of studying experience is described by Smith and Osborne [22] as a double hermeneutic, which can be useful for discovering meaning which may be hidden due to the phenomena’s mode of appearing [24].

## Methods

Within the iterative project design, there were two distinct phases of data collection. The first data collection phase involved conducting focus groups with the following stakeholders: problem gamblers in treatment (focus group 1), gaming venue staff (focus group 2), consumer advocates with lived experience of PG (focus group 3) and gambling help service counsellors (focus group 4).

Focus groups are used to collect specific types of information from clearly identified groups of individuals [25]. They have an advantage over individual interviews in that the group setting provides a more social environment, as participants influence and are influenced by others—as they are in real life [26]. This is particularly useful when the phenomena of interest involve individuals making decisions that are made in a social context. For instance, a decision by venue staff to approach a patron and initiate referral to a gambling help service is made involving discussion with other staff. Therefore, examining this process in a group setting provides a useful medium to obtain rich and valid data. An iterative process was employed whereby data from each focus group were analysed, before subsequent groups were conducted. Data from former groups were revisited before moving onto the next, and emerging insights helped inform the semi-structured questions used for the focus groups that followed. Whilst focus groups do have some limitations [26], it was decided that this was the best way to explore initial themes, which could be followed up by in-depth interviews.

In line with the iterative nature of the study, findings from the focus group analyses then helped to determine the most important questions to follow up in the in-depth interviews conducted in the second phase of data collection with problem gamblers. This provided an opportunity to follow up on emerging themes and insights and to examine these from other target group perspectives, in addition to uncovering new themes as they emerged.

The second data collection phase involved conducting a series of semi-structured individual in-depth interviews with the following two groups: Aboriginal and/or Torres Strait Islander individuals impacted by PG, problem gamblers attending PG counselling. These two groups were purposefully chosen based on themes that had emerged from the stage 1 focus group analyses. The aim of this second phase of data collection and analyses was to investigate emerging themes in greater depth.

In-depth interviewing involves conducting intensive individual interviews with a small number of participants to explore their perspectives on a particular idea, programme or situation [27]. Interviews were semi-structured to allow the interviewers some control over the direction of the content to be discussed, whilst allowing participants to elaborate or take the interview in new but related directions [27].

### Target population and sampling techniques

A mixed purposeful sampling method combining three different strategies (maximum variation, expert, homogenous) that were considered most consistent with the research purpose was used to recruit participants across all focus groups and in-depth interviews. Purposeful sampling is used in qualitative research to select information-rich cases related to the phenomenon of interest [28]. The purpose of this method of sampling is to gain a deeper understanding of the phenomena of interest, rather than to generalise findings to a wider population [29].

Table 1 presents the target population, sampling techniques and procedure for the four focus groups. A purposeful maximum variation method was used for groups 1 and 4 (problem gamblers, counsellors) to gain a wide range of age, gender and sociodemographic variation among problem gamblers, along with a group of counsellors from a range of different organisations. Problem gamblers were current clients of two local gambling help services. A senior staff member of each service contacted suitable individuals and invited them to participate in the study. To recruit counsellors, managers of six distinct gambling help services approached suitable participants and invited them to participate in the study. For groups 2 and 3 (venue staff, consumer advocates), purposeful expert sampling was used, to target venue staff with experience in approaching potential problem gamblers, which are predominantly the managers of gaming venues, and a local consumer advocate group was targeted to access individuals with lived experience of PG who had good knowledge of local gaming room policies and procedures. To recruit venue staff, a representative from the Australian Hotels Association approached suitable individuals and invited them to participate in the study. All interview guides were semi-structured. Groups 1 and 3 focused on gamblers’ journey to help-seeking, experience with venue staff interacting with them around PG, experiences with the Gambling Help Line and experiences with gambling help materials in the venue. Group 2 (venue staff) focused on experience with interacting with patrons of concern, experience with available responsible gambling materials in the venue and experience with interacting with patrons of culturally diverse backgrounds in concerning PG. Group 4 (counsellors) focused on experience of PG clients talking about their interactions with hotel staff, experience with the available support and help materials in the venues and experience regarding their clients’ use of responsible gambling material in venues. Prior to conducting the focus groups, two researchers spent time in the field observing the gambling venue environment and interactions between staff and patrons. Reflective memos were made which were later used to enhance and inform the interpretive analysis process. A dynamic analysis process was achieved with observation of environment and participant interactions, reflection by both researchers in situ and analysis of the actual transcribed data. Table 2 presents the final makeup of the focus groups. Each focus group was conducted by two researchers: one facilitated the interview; the other operated the recording device and took detailed observational notes which included any remarkable pauses, gestures and speech dynamics [30]. Immediately following each focus group, the two researchers discussed any notable elements of the group content and their own experiences. These debriefing sessions were digitally recorded and professionally transcribed and incorporated into the analyses. Based on the initial analysis of focus group data, both purposeful maximum variation and purposeful homogenous sampling were used to recruit participants for the in-depth interviews. From the focus group data, venue staff and counsellors spoke of challenges they experienced engaging with Aboriginal gamblers. This was an issue we wished to follow up; hence, we wanted good variation among problem gamblers in treatment along with targeting a number of First Nations people with lived experience. Five participants of Indigenous Australian background were purposely selected, and a further six participants were recruited from two metropolitan-based PG help services via counsellors. The semi-structured questions for the in-depth interviews explored participants’ journey to help-seeking; the influence of important people in the participant’s decision to seek help; the influence of participants’ cultural background on their decision to seek help; participant’s experience with responsible gambling messaging, particularly concerning their journey to help-seeking; and experience of venue staff concerning help-seeking.

**Table 1:** Target population, sampling techniques and procedure for focus groups

Focus group	Target population	Sampling technique	Location	Group facilitator	Group observer
1	Problem gamblers in treatment	Purposive maximum variation	University campus conference room	PhD student experienced with focus groups	PhD student experienced with PG counselling
2	Gambling venue staff	Purposive expert	Hotel boardroom central to city	PhD student experienced with focus groups	PhD student experienced with PG counselling
3	Consumer advocates with lived experience of PG	Purposive expert	Meeting room at local GHS	PhD student experienced with focus groups	PhD student experienced with PG counselling
4	GHS counsellors	Purposive maximum variation	University campus conference room	PhD student experienced with PG counselling	Senior research fellow experienced with focus groups

*GHS* gambling help service

**Table 2:** Sample characteristics of 32 focus group participants

Characteristics	Participants
Focus group 1: problem gamblers, *n* = 8	
Gender	
Male	2
Female	6
Age	
18–39	0
30–39	7
40–49	1
50–59	0
60+	0
Length of time having a gambling problem	
Less than 12 months	1
1–2 years	1
2–5 years	1
5–7 years	1
7–10 years	1
10+ years	3
Marital status	
Married/de facto	2
In a relationship	1
Separated/divorced	2
Widowed	0
Single	3
CALD background?	
Yes	2
No	6
Employment status	
Employed full time	6
Employed part time	1
Unemployed	0
Retired	0
Home duties	0
Disability pension	1
Student	0
Focus group 2: venue staff, *n* = 10	
Age	
18–39	1
30–39	5
40–49	1
50–59	3
60+	0
Length of time working in a gaming venue	
Less than 12 months	0
1–2 years	0
2–5 years	0
5–7 years	1
7–10 years	3
10+ years	6
Marital status	
Married/de facto	2
In a relationship	3
Separated/divorced	1
Widowed	0
Single	4
CALD background?	
Yes	0
No	10
Employment status	
Employed full time	7
Employed part time	0
Employed casually	3
Focus group 3: consumer advocates, *n* = 7	
Gender	
Male	4
Female	3
Age	
18–39	0
30–39	0
40–49	4
50–59	3
60+	0
Length of time having a gambling problem	
Less than 12 months	0
1–2 years	0
2–5 years	2
5–7 years	1
7–10 years	1
10+ years	3
Marital status	
Married/de facto	6
In a relationship	1
Separated/divorced	0
Widowed	0
Single	0
CALD background?	
Yes	3
No	4
Employment status	
Employed full time	1
Employed part time	3
Unemployed	0
Retired	2
Home duties	0
Disability pension	1
Student	0
Focus group 4: problem gambling counsellors, *n* = 7	
Age	
18–39	0
30–39	4
40–49	2
50–59	1
60+	0
Length of time working as a problem gambling counsellor	
Less than 12 months	1
1–2 years	1
2–5 years	2
5–7 years	1
7–10 years	1
10+ years	1
Marital status	
Married/de facto	6
In a relationship	1
Separated/divorced	0
Widowed	0
Single	0
CALD background?	
Yes	3
No	4
Employment status	
Employed full time	5
Employed part time	2

*CALD* culturally and linguistically diverse

In total, 11 in-depth interviews were scheduled with individuals with lived experience of PG. Nine interviews were conducted as two individuals did not attend their scheduled interview. These individuals were subsequently followed up and, in order to accommodate their work schedules, offered phone interviews but they declined to participate. The interviews were conducted by the PhD students who collected the data in phase 1. The interviews were carried out at locations convenient for the participants and included a university office, the office of a PG help service and the two specialist services. Table 3 presents the characteristics of individuals who participated in the in-depth interviews.

**Table 3:** Sample characteristics of nine in-depth interview participants

Characteristics	Participants
Gender	
Male	4
Female	5
Age	
18–39	0
30–39	1
40–49	3
50–59	3
60+	2
Length of time having a gambling problem	
Less than 12 months	0
1–2 years	1
2–5 years	3
5–7 years	2
7–10 years	2
10+ years	1
Marital status	
Married/de facto	3
In a relationship	1
Separated or divorced	2
Widowed	0
Single	3
CALD background?	
Yes	6
No	3
Employment status	
Employed full time	1
Employed part time	1
Unemployed	0
Retired	1
Home duties	2
Disability pension	4
Student	0

*CALD* culturally and linguistically diverse

### Data analysis and verification procedures: revealing the phenomenon

With participants’ consent, all interviews were digitally recorded. Recordings were then professionally transcribed and checked for accuracy by two researchers. Due to a technical failure, one in-depth interview audio recording was not made, and thus, a transcript was not produced. This failure was detected immediately after the interview was complete, and thus, the researcher was able to make detailed field notes about the specific content of the interview, including verbatim quotes. Transcripts were uploaded into NVivo 11 qualitative data software tool [31]. In line with the two phases of data collection, there were two corresponding phases of data analyses. The analytical guidelines for IPA as recommended by Pietkiewicz and Smith [23] were applied. Firstly, audio recordings from the focus groups were listened to and the verbatim transcriptions read multiple times independently by two researchers. At this stage of the analysis, the focus was directed to what the text was saying [32]. Individually, the researchers made initial interpretive exploratory notes during readings which they transformed into potential emergent themes. The researchers then met to examine connections between potential themes and group them together according to conceptual similarities. The same process was then applied to the phase 2 in-depth interview dataset. Next, all transcripts were phenomenologically coded and phenomenological clusters developed. Data from the group facilitators’ debriefing sessions and observational notes were included at this stage of the analyses, to assist with interpretation of the text. As the text was read and re-read, individual parts of the text were interpreted in the context of the whole dataset. Continual movement between the parts and the whole (the hermeneutic cycle) led to a deeper understanding of the text and allowed us to move from an understanding of what the text was saying to understanding of what it talked about. ‘The sense of a text is not behind the text, but in front of it. It is not something hidden, but something disclosed’ [32], p87. For example, text noting how venue staff were fearful of a negative response from gamblers if they approached them with concern was initially interpreted as a fear of gamblers who may be angry about losing their money. Further engagement with the text, supported by observation and reflection on participants’ expressions and body language, and the researchers’ interpretation of what these processes meant however, resulted in a deeper understanding of the aforementioned fear. The insight that emerged was that the fear stemmed from staff perceiving they were being hypocritical by encouraging patrons to gambling on the one hand and then discouraging them if the gambled too much, and gamblers were aware of this perceived hypocrisy and could react adversely. This careful analytical process led to a final list of interpretive superordinate and subthemes. Following the analyses of the focus group and in-depth interview data, findings were combined to enhance data richness. Use of such methodological triangulation provided diverse ways of looking at the same phenomena and enhanced credibility by strengthening confidence in conclusions derived [33, 34].

## Results

The characteristics of the sample for the focus groups and individual interviews are shown in Tables 2 and 3. A total of 41 participants (13 male) comprised four separate focus groups and nine individual in-depth interviews. The majority of participants (68%) were aged between 30 and 49 years and either married, de facto or in a relationship (64%).

### Themes emerging from the data

Initial readings of the interviews provided the impression that harm reduction policies in the gaming room created a particularly stressful environment for staff. Furthermore, gamblers (who were largely unaware of such policies) and counsellors did not believe it was in the venues’ interest to implement them rigorously. Early in the analysis, an enormous amount of data was produced concerning individual experiences and perspectives. Through application of the hermeneutic cycle (with rich discussion supported by detailed reflective memoing by the two researchers and further debate and discussion by the broader research team to support rigour in data interpretation), the following insights emerged. Although subjective experiences of the phenomena were presented differently in the text, shared meanings were uncovered across the focus groups and individual interviews. Six themes were revealed through the focus group data which were supported by the in-depth interviews. The in-depth interview data led to the extension of one theme and the emergence of another, resulting in seven themes. Most notably, perceived stigma significantly influenced gamblers’ help-seeking behaviour, which tended to be when they had reached crisis point. A personal connection with trusted venue staff was paramount for an effective interaction around PG. The perceived divergence of gaming room staffs’ hospitality duties and responsible gambling obligations was a discrepancy experienced from many perspectives across focus groups and in-depth interviews and hindered interactions between gamblers and staff. As the purpose of this research was to examine the help-seeking experience in the context of gambling venues from several perspectives, quantification (e.g. frequency counts) was not undertaken. To illustrate the themes that emerged, relevant examples from the discourse are presented.

#### Personal connection

Personal connection related to the importance of rapport between problem gamblers and gaming venue staff, gaming venue staff and gambling help service staff, and problem gamblers and gambling help service staff. A genuine and personal connection was crucial between venue staff and patrons for both the staffs’ willingness and confidence in initiating engagement with patrons around their level of gambling and for acceptance of such interactions by patrons. In addition, a personal relationship was highlighted as being of principal importance between staff of gaming venues and gambling help service staff. A close working relationship between the two parties was viewed as essential for creating an environment in which effective referrals could take place. Such a relationship would also provide support for venue staff and compliment the work of the hotel and club’s responsible gambling early intervention agency teams. Rapport was generally built over time, as indicated by the following participant response:

I wouldn’t be here today if it wasn’t for one of the people in the venues. She didn’t approach me outright. She just kept an eye on me, and she’d come around, just quickly, say, ‘Oh how are you today?’ and slowly I got to know her, and with the problems I had I started to confide in her, and on my own bat, I rung up the help, and that’s how the staff, I couldn’t thank her enough, because it changed my life. But like I said, the venue people, they try, but all depends how you click with them, I think (Female participant, focus group 1).

A close working relationship between gaming venues and gambling help services would also provide opportunities for feedback for staff regarding outcomes of patrons referred to help services, which was reported as one factor that might further encourage referrals. The personal connection theme also related to PG participants’ negative experiences with the national gambling telephone helpline and the lack of information about local support services. This was particularly relevant for culturally diverse populations, though a number of Aboriginal participants explained that they would be reluctant to seek help regardless of a good relationship with staff or awareness of services:

We’re supposed to be strong in culture, strong Aboriginal women, and we do it all on our own. We grew up to be like that (Female participant, in-depth interview).

#### Role conflict

A perceived conflict between venue staffs’ hospitality duties and responsible gambling obligations was a discrepancy experienced from many perspectives across all groups. Though not visible initially, multiple readings of the text moving continuously between the parts to the whole allowed this theme to be revealed across the whole dataset. Venue staff described experiencing a distinct conflict between expectations to create a comfortable environment for patrons to gamble in, whilst at the same time being mindful of their obligations to monitor patrons’ spending and to intervene if necessary. This issue received much attention in the focus groups and was clearly a source of stress for venue staff, who at first were reluctant to talk about it. It took around 15 min of informal discussion and long periods of silence before staff began to speak openly about this issue. Once they began, however, their tone became more animated and pronounced as they heard other group members share similar experiences. Participants were visibly torn, frustrated and agitated when describing harm reduction in the context of their duties. Staff were conflicted with the divergence between business and care, as illustrated by the following exchange:

There’s a very fine line between running a business and caring about your patrons. And I think that’s the hardest thing with every person that works in gaming, is that if you remove a big punter and your senior sees that, they go ‘what are you doing, you’re ruining our income’. Well hang on, I have a duty of care to my patrons, where’s the line? (Female participant, focus group 2).

Problem gambling participants described being acutely aware of such conflict of roles, which led to any approach by concerned venue staff, being perceived by gamblers as disingenuous or hypocritical. Though many of the gamblers spoke about their desperation for help, they clearly viewed venue staff as promoters of gambling, which significantly hindered any well-meaning harm reduction interactions. The researchers noted the resentment from the gamblers during focus groups 1and 3 (gamblers) when venue-driven harm reduction strategies were discussed and made the comment that gamblers appeared to take particular offense to the perceived hypocrisy.

They’ve got a conflict of interest. It doesn’t work if its direct staff. You’ve got management that – they want to keep people there that spend big money. There is a conflict. (Female participant, focus group 1).

Yeah. You know that they don’t care. (Male participant, focus group 1).

This perceived hypocrisy also impacted on participants’ preference regarding help-seeking, which was best exemplified by the following comments:

Well it’s not to the pub’s advantage to tell people to leave and not gamble, is it? For me, I wouldn’t use it (gambling help information and support) if it was in the pub. It seems almost hypocritical, you know what I mean? (Male participant, in-depth interview).

Counselling staff also described their experience interacting with venue staff around role conflict.

I’ve had a few come up and take my card and rang and said that they were quite stressed about the sort of position that they were in around having to have these responsible gambling checks, but also not being able to do a lot of the reporting in the way like perhaps approaching people for reasons around not feeling that they would get the backing of management. So I think people (venue staff) learn to manage the stress of working in that really conflicting role that they’re in by turning a blind eye. (Female participant, focus group 4).

An initial reading of the following comment gave the impression that staff viewed heavy gamblers as ‘out of control addicts’ who could become aggressive if interrupted. However, re-reading the text, moving between the parts to the whole, revealed that staff felt they were constantly shifting between being a hospitality worker and a counsellor or private investigator, with the latter raising the risk of an adverse response from patrons. Venue staff described feeling they were being forced to make a moral judgement in identifying patrons who were deemed to be overspending. This influenced their reluctance to approach patrons and at times involved a level of fear concerning how patrons might respond.

But then at what point did our job descriptions include private investigator?

(Female participant, focus group 2).

So I feel that even though we do record and we do speak to them, you’ve got to be careful want you do say and when you do speak to them, because quite often they can be quite aggressive. And you’re putting yourself into danger in that reason. I’ve come up to a few aggressive ones. (Female participant, focus group 2).

Venue staff explained they would feel much more comfortable and willing to provide responsible gambling-related educational material (information about the nature of gaming machines, details of available help services) to all patrons irrespective of their level of gambling, as it removed the need to make a perceived moral judgement. The following comment illustrates this sentiment which was echoed by gamblers in focus groups 1 and 3.

Maybe like if I was trained and I went up to every customer and said ‘I’m the duty manager here, I’ve been trained, this is my new responsibility to advise everybody of the service available if you ever feel like you have a problem’. And if you’re doing it to everybody, no one is going to feel singled out. So that could kind of get rid of a bit of that issue where if you go up to somebody. (Female participant, focus group 2).

#### The tipping point to help-seeking is individualised

The majority of PG participants indicated their help-seeking was associated with a point of crisis, such as loss of employment, getting ‘caught’ or the gambling problem becoming disclosed or going through a divorce. Help-seeking at this point typically involved phoning the Gambling Help Line or approaching venue staff to initiate a self-barring order. This theme was also based on the shared experience among venue staff of the tendency to initiate contact with a patron of concern only if they displayed overt signs of distress. An initial reading of the following comment gave the impression that staff believed at times feeling as though the entire gaming room was full of problem gamblers and that it was not feasible to intervene with them all. However, through a deeper engagement with the text, it became clear staff found themselves in a difficult position in that they were required to consider which gamblers could afford to lose their money. This was described as a highly individualised and private matter. Staff also felt pressure in making a moral judgement and their concerns about incorrectly approaching a patron who did not have a problem, that is, making a false positive identification. Consequently, they tended to wait for overt and/or disruptive behaviours before initiating an approach.

I think the room is full of a lot of problem gamblers, but then I think it’s easier to identify when there’s a real, real problem. Do you know what I mean? I think we’re surrounded by problem gamblers. (Female participant, focus group 2).

Because she’s a doctor, and she comes in after – she obviously works all night and then she comes in first thing in the morning when she’s finished…everyone’s done like a gaming report on her, and we do believe what she says is true, that she does have enough money. (Female participant, focus group 2).

Through the in-depth interviews, it emerged that what constituted a motivator for help-seeking was different for each person and that the ‘tipping point’ existed on a continuum. The majority of in-depth interview participants described accessing help well before experiencing a significant crisis. One male participant reported accessing help when ‘I was getting close to my moral line’. Some participants explained that they were dismissive of gambling help services and responsible gambling material, as they did not view themselves as problem gamblers. However, some of these same individuals reported accessing help at some point when information was offered, before reaching what might be described as a significant crisis. As such, some individuals though not acknowledging they might have a problem chose to access help when information was offered at various points prior to reaching a state of crisis. Such points included accessing help for co-morbid mental health and substance use issues.

#### Discretion and privacy

Across all focus groups, discretion and privacy were considered paramount in the effective facilitation of help. This related to both the timing and location of interaction between venue staff and patrons and to the physical placement of responsible gambling messaging and gambling help service information within the gambling establishment. Accessing gambling help information was described as an extremely difficult thing to do. One gambling counsellor reported ‘I’ve had clients tell me that it’s too embarrassing to pick up information, that they wouldn’t do it’. Problem gambling participants indicated the responsible gambling materials in the gaming rooms were largely ineffective, because they were not in a clear frame of mind whilst gambling. A number of participants spoke of signage they had seen in the bathroom and explained that this particular material had been effective for two reasons. Firstly, they were able to read the information in a private place. Secondly, they were away from the gaming room and in a clearer mindset, which allowed them to be more open and receptive of responsible gambling information. The following exchange by group members reveals this point:

What has made me think about my gambling when I’ve been gambling, is if they’ve had a large advert of seeking help or what are you doing? The men’s toilets had a sign on the door as you go in or as you come out. I think there was even one over the urinal from memory, if I could use that expression, and it did make me think what I was doing. (Male participant, focus group 1).

The stuff that’s in the gaming room, I bet you it’s there, but we don’t see it, or we didn’t see it, because you deny. You’re in denial for a long time. (Female participant, focus group 3).

When you go sit down on the toilet and shut the door, it’s the same as females with pap smears (laughs). (Female participant, focus group 1.)

You’re away from the machine so you’ve got time to read something. (Male participant, focus group 1).

Privacy was of particular importance among Aboriginal participants, who described a reluctance to seek help under almost any circumstances. This was due to both significant shame and embarrassment and a belief that they were responsible for their problem and should therefore be responsible for resolving it alone.

We don’t like to put ourselves out there. If we’ve done something wrong, we don’t want to have to admit that, I suppose. Aboriginal people, we do get really embarrassed about stuff like that, but that’s why we don’t always say stuff. We just keep it close to us because we’re ashamed of it. (Aboriginal Male participant, in-depth interview).

Shame and embarrassment were very common experiences among gamblers and resulted in them keeping their problems secret. As the text was read and re-read with continuous movement between individual responses and the dataset as a whole, what emerged was an understanding that privacy had a particular effect for Aboriginal respondents. Not talking to anyone about their problem led to a feeling of isolation and a perception that they were the only ones that felt ashamed. This is best exemplified by the following comment:

I think Nunga people think that they are the only ones that feel that way but they’re not the only ones. Just like when white people first came here, they thought we were animals. We all feel the same way. It’s not until 150 years later where we are acknowledged as human beings. We are just the same, we just feel like we are the only ones that are ashamed of behaviour. (Aboriginal female participant, in-depth interview).

#### Organisational inconsistencies

Problem gambling participants expressed frustration that they could self-bar themselves in one venue then walk across the road to another and continue gambling. This was echoed by venue staff who expressed frustration that due to privacy issues, they could not share information about patrons of concern with neighbouring venues. This led to a feeling of apathy among venue staff in that barring or approaching a patron of concern around their excessive gambling may not in fact reduce any harm, as the patron may merely attend another nearby hotel.

Because like this lady – well most of our customers, if they’re barred, what they’re going to do is walk down the end of the road, cross over and there’s another one (Male participant, focus group 2).

She’s just down the road. She’s like 200 metres down the road at the next pub. And it’s like, well it’s just ridiculous. (Female participant, focus group 2).

In these above two comments, it was clear that the participants were frustrated. They had previously discussed how stressful it was to approach a patron of concern about their level of gambling. They explained that even if they did raise the courage to engage the gambler, the gambler would just go and continue gambling at another venue.

Problem gambling participants suggested that multiple and frequent non-threatening approaches by concerned staff across numerous venues would be more effective in encouraging them to seek help, even if the approaches were not received well at the time, than a penultimate contact initiated by a ‘red flag’ incident when a patron displays overt signs of distress. The ‘inconsistencies between organisations’ theme was also based on the experiences of PG help service staff who reported that some agencies were much more active in fostering relationships with local gambling venues than others. Also within this theme was the inconsistency between venues in the type and manner of PG help service information that was made available to gamblers. The organisational inconsistencies theme was supported also via in-depth interview data, regarding the inconsistent application of barring orders by venues. One participant living in an inner rural city discussed their experience of one venue being highly vigilant in upholding the barring order and refusing entry and the opposite experience in another venue close by.

#### Lack of awareness

Problem gambling participants and gaming venue staff expressed a general lack of awareness of the available support services for individuals struggling with gambling issues. Other than the Gambling Help Line, participants were largely unaware of the range of available specific services and the nature of the assistance they provided. This theme was also based on PG participants’ lack of awareness of the responsible gambling training that venue staff must undertake. In addition, gamblers were unaware of venue staffs’ obligations around monitoring patrons’ spending habits and their duty to identify potential patrons of concern and intervene as necessary. In fact, one participant reported their lack of awareness that staff were able to offer a suite of options for patrons experiencing difficulties with their level of gambling and was of the belief that venue staff could only assist by facilitating a barring order. This reduced the likelihood of them approaching venue staff for help.

I didn’t know how staff would be able to help me, and the only way that I thought that they could help me was to bar me, and I didn’t want to be barred. (Female participant, focus group 1).

A number of other participants agreed with this and noted that this attributed to patron reticence to approach staff, unless they wanted to action a self-barring order.

Now, nowhere in the gaming venues does it tell you the staff have the information to direct you to the right organisation. (Female participant, focus group 1).

#### Relapse: a hidden and common experience

This theme emerged through a deeper understanding of the help-seeking process which emerged through the in-depth interviews. With insights gained through these interviews, we re-engaged with the focus group text and continued the interpretive process. It became clear that many gamblers made the decision to stop gambling well before they accessed formal help. They made many attempts to stop and saw each relapse as a failure, which ironically, made them less likely to seek help. A number of participants explained that although they had accessed help at some point, they were very reluctant to access help again following a relapse of their gambling behaviour. One female participant explained:

I have had the help before and it’s great but I think you sort of feel like you don’t want to go back there because you’ve broken your own sort of rules.

There was significant shame around relapse, with one participant stating they were surprised when they learned that many problem gamblers relapse. The shame and self-stigma around perceived failure inhibited appropriate help-seeking. This is highlighted by the following participant’s statement:

Well, you’ve got to face the person that you’ve sat with for quite a bit of time and discussed it with and you know in your own head that everything you’ve said is right, you know, and no, it’s not comfortable. It doesn’t feel comfortable coming back and saying, ‘Hey, I played the pokies again. I failed’ you know? (Male participant, in-depth interview).

## Discussion

This study aimed to examine the experience of identification and response to PG in venues, through qualitative analysis of the perspectives of problem gamblers, gambling venue staff and gambling counsellors. The results of this research may provide an insight into how gambling venues could effectively help facilitate help-seeking among problem gamblers. That being said, notably, the findings also seriously question whether gambling venues and their staff are in fact a suitable means for harm reduction. Given that almost half of gaming machine revenue is generated from problem gamblers, this is an important question and we hope this study will encourage researchers and policy makers to explore this further.

The findings from the current study suggest that venue staff approach patrons of concern predominately when they exhibit significant visible overt PG behaviours. This is consistent with findings from previous research [17, 35]. The data suggests three main reasons for this, all which sit within the theme of role conflict.

Firstly, despite venue staffs’ reported confidence in their ability to identify a potential problem gambler, they are particularly reluctant to overtly make what they perceive to be a moral judgement about a patron. This includes staffs’ reservations about making incorrect assumptions about a patron’s ability to support their gambling, irrespective of whether they are based on a set of observable indicators. It also includes staffs’ fears of a negative response such as anger.

Secondly, the conflict staff experience between their dual roles of facilitating the use of gaming machines in the context of a commercial business and their obligations to ensure patrons do not gamble excessively, create a perceived dilemma. This is particularly difficult in situations where staff feel unsupported by upper management. This dilemma results in staff directly engaging with patrons of concern, primarily only when they become visibly distressed or disruptive. In spite of continuing improvements in venue staffs’ confidence in identifying problem gamblers, an aversion by staff to target or single out a patron and share their concern (e.g. by providing information and/or referral to treatment) appears to be an important barrier to the dissemination of responsible gambling and treatment service information to problem gamblers.

Thirdly, venue staff appear to become desensitised to the extent of patron spending and prevalence of PG in venues. Again, this results in them identifying and responding to patrons of concern chiefly only when they display significant, clear and overt PG indicators.

Role conflict experienced by venue staff was a key theme in the current data and has been described in previous research as a source of stress among staff [9]. The current data suggests that not only is role conflict a source of stress for venue staff, it also affects their willingness to directly engage with problem gamblers about their gambling. Furthermore, the findings in the current study that problem gamblers also experience the role conflict described by venue staff and that this perceived hypocrisy inhibits their receptiveness of staffs’ interactions with them around their level of gambling, are new and to our knowledge have not been previously reported. Role conflict as described in this study, both for venue staff and problem gamblers, appears to have an important influence on effective engagement between the two parties concerning the provision of responsible gambling information and referral to gambling help services.

Overall, problem gamblers found the gaming room to be an unsuitable location to engage with help messaging due to their state of mind whilst gambling (e.g. ‘in the zone’). Discrete areas close to, but not within, the gaming room were seen to be appropriate for the display of responsible gambling messaging and help materials. These findings suggest that it could be helpful if gamblers could access this information privately and in a context that supports self-reflection and/or personal engagement with the material for example bathroom doors, the gaming room foyer and designated smoking areas of gambling venues.

Gambling venue staff were confused and internally conflicted with respect to their responsible gambling obligations, limiting the quality and frequency of interactions with potential problem gamblers. At the same time, problem gamblers demonstrated limited awareness of the responsible gambling training gaming venue staff undertake as part of their role, which in turn contributed to their reluctance in engaging with staff around help-seeking as they did not perceive gaming venue staff to be potential sources of help. To overcome these inhibitors, venues could consider training for gaming venue staff that encourages a greater focus on the provision of responsible gambling information to all gamblers, rather than solely engaging with identified patrons of concern. Providing such information to all gamblers may help to eliminate the current perception that staff are required to make moral judgements about a patron’s level of risk to harm, which has been associated with staff reluctance to approach patrons and refer to gambling help services, whereas provision of harm reduction material as a matter of course creates an environment conducive to the non-judgemental and open exchange of responsible gambling education and support. The outcome of adopting such an approach across all gambling venues is that all patrons will come to expect a dialogue around responsible gambling practice and available support services at some point. The caveat here, of course, is that the present findings indicate gamblers do not view venues as potential sources of help and perceive venue-based harm reduction initiatives to be insincere.

The findings from this study have a number of limitations which should be considered. The samples are not representative in any way of venue staff, problem gamblers or counsellors and so cannot reveal anything about the prevalence of such experiences. Furthermore, the samples are all Australian and the views expressed may not represent those from other jurisdictions. All problem gamblers in this study had accessed treatment at some point, and therefore, their views may be different from non-treatment-seeking problem gamblers or gamblers with less severe problems. The views of non-problem gamblers and non-help-seeking problem gamblers could be explored in further research.

## Conclusion

In summary, harm reduction materials in gambling venues could include personalised local help service information rather than a generic national help line, particularly for culturally diverse populations. Special attention should be paid to developing effective harm reduction and engagement strategies for Aboriginal gamblers, who are exceedingly reluctant to seek help. That being said, problem gamblers do not view the gambling venue as a place to access help-seeking information, in part due to the business versus care paradox and also because they are unaware of staffs’ harm reduction training and obligations. The results from this study indicate that the involvement of venue staff in the help-seeking process is complex, stressful, conflicted and often ineffective for both staff and gamblers. The paradox between venue staff promoting gambling whilst discouraging excessive gambling is a conflict experienced by both gamblers and staff and appears to be particularly detrimental to effective engagement between the two. This conflict needs to be taken into account when considering harm reduction strategies in gambling venues. Moreover, the fundamental notion that gambling venues (which are driven by profits) are suitably able to implement and regulate harm reduction policies requires further exploration.

## Abbreviations

IPA: Interpretive phenomenological analysis
PG: Problem gambling
